# Respiratory Failure due to Severe Obesity and Kyphoscoliosis in a 24-Year-Old Male with Molecularly Confirmed Prader-Willi Syndrome in Tertiary Hospital in Northern Tanzania

**DOI:** 10.1155/2017/2348045

**Published:** 2017-04-09

**Authors:** Elichilia R. Shao, Lucas F. Kiyegi, Amos O. Mwasamwaja, Kajiru Kilonzo, Ben C. J. Hamel

**Affiliations:** ^1^Department of Internal Medicine, Kilimanjaro Christian Medical Centre, P.O. Box 3010, Moshi, Tanzania; ^2^Department of Internal Medicine, Kilimanjaro Christian Medical University College, Tumaini University Makumira, P.O. Box 2240, Moshi, Tanzania; ^3^Better Human Health Foundation, P.O. Box 1348, Moshi, Tanzania; ^4^Imagedoctors International, P.O. Box 16341, Arusha, Tanzania; ^5^Department of Human Genetics, Radboud University Medical Center, Nijmegen, Netherlands

## Abstract

Obesity, mild intellectual disability, hypotonia, poor sucking, cryptorchidism in males, hypogonadism, and kyphoscoliosis are common features of Prader-Willi syndrome (PWS). We report a case who had severe respiratory complications due to extreme obesity and kyphoscoliosis, which are important causes of morbidity and mortality, and discuss management. Furthermore, this is the first molecularly confirmed PWS case in Sub-Saharan Africa outside South Africa.

## 1. Introduction

Prader-Willi syndrome (PWS; OMIM 176270) is a well-known complex genetic condition caused by a deficiency of paternally expressed genes in the chromosomal region 15q11–q13, due to a de novo deletion in the paternally derived chromosome 15 in 70–75%, while the rest is due to maternal uniparental disomy (25%), unbalanced translocations, and genomic imprinting defects, all involving chromosome 15. Conversely, loss of maternally expressed genes in the chromosomal region 15q11–q13 will bring about another entirely different clinical disorder called Angelman syndrome. PWS has a prevalence of 1 : 10,000 to 1 : 30,000 individuals [1, 2]. A dysfunctional hypothalamic-pituitary axis is associated with clinical features such as hyperphagia, deranged hypoxic ventilator function, and hypogonadism. Endocrine problems like diabetes mellitus are common due to abnormal glucose tolerance and insulin resistance. Other characteristics include severe hypotonia observed at birth and during neonatal period, while gradual feeding difficulties, thick saliva, lethargy, mostly mild intellectual disability, and small genitalia with cryptorchidism in males become apparent. Dysmorphic features such as narrow bifrontal diameter, small feet and hands, and almond shaped palpebral fissures are seen. In older children and adolescents growth is delayed as is development of secondary sexual characteristics. Later in life patients may also present with characteristic behaviour such as skin picking, temper tantrums, and obsessive-compulsive features [1, 2]. In a recent review it was argued that in patients of 13 years and older cognitive impairment, excessive eating, hypogonadism and/or typical behaviour problems, and short stature should prompt DNA testing for PWS [1]. The excessive eating due to hyperphagia and/or obsession with food contributes to significant weight gain, which together with musculoskeletal deformity, like kyphoscoliosis and deranged hypoxic ventilator function, can lead to severe respiratory problems [3, 4].

We report a molecularly proven PWS case with respiratory failure due to obesity and kyphoscoliosis and discuss its management.

## 2. Case Report

A 24-year-old man was referred to our hospital due to on and off difficulties in breathing with excessive eating. He was born vaginally after an uneventful, full term pregnancy weighing 2.6 kg (~3rd centile). Soon after delivery he was floppy and had a weak cry and difficulty in sucking. His milestone development was delayed as he started sitting at 15 months and walking at 25 months. At the age of 25 months he started eating excessively and gaining weight. He also had night snoring and episodes of difficulty in breathing (DIB). At 3 years he developed chest deformity and back swelling (neck bump). He started school when he was 7 years but stopped because of poor performance and fighting with other students because they were making jokes at him about his neck bump. He showed abnormal behaviour like food obsession and irritability. When he was 10 years the DIB further progressed and he was treated at a local health centre without any improvement. The swelling at his back progressed when he was 20 years old when he developed obstructive sleep apnoea (OSA) which worsened as BMI increased. There is no history of smoking or taking alcohol. He is the first born in the family of seven children, five boys and two girls. The second born is 22 years old and he is in college while the last born is 7 years old and in primary school. No other member of the family has the same or similar problem. Father is a plumber while the mother is the housewife. At 24 years DIB was accompanied by general body malaise and he was referred to Kilimanjaro Christian Medical Centre (KCMC) consultant hospital for expert management. Up to then no etiological diagnosis was reached and no surgical interventions took place for cryptorchidism nor kyphoscoliosis.

**Figure 1:**
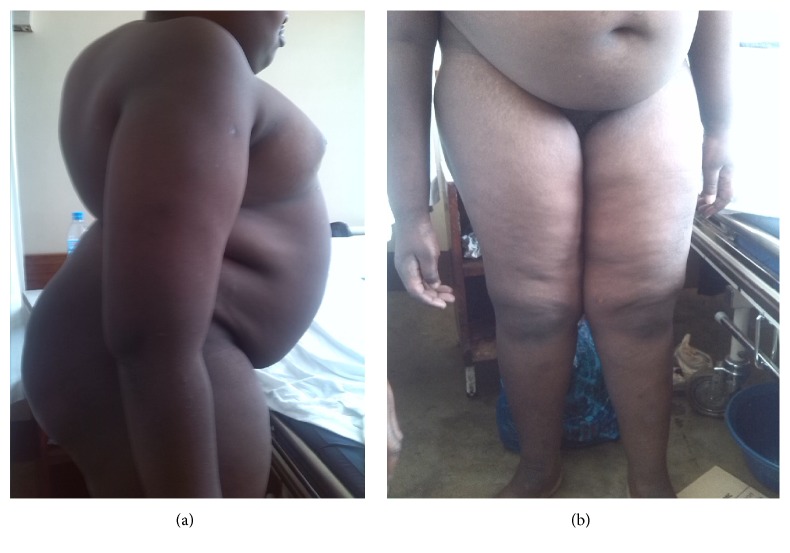
The clinical photos showed Kyphoscoliosis and truncal obesity (a) and poor developed genitalia (b) in a 25-year-old male with Prada-Willi Syndrome.

On physical assessment height is 132 cm (far below 3rd centile), weight 65 kg (for height: above 97th centile), and body mass index (BMI) 37.3 kg/m^2^ (>97th centile; gross obesity). His head circumference was 53.3 cm (10th–25th centile) [5]. The most obvious dysmorphic features were narrow bifrontal diameter and downturned corners of the mouth. He also has very short hands. Furthermore, he had a webbed and short neck and rotated thorax due to kyphoscoliosis (Figure 1(a)). He also had poorly developed secondary sexual characteristics such as few dispersed pubic hairs and bilateral empty scrotal sac with very small penis measuring 2.4 cm (far below 3rd centile) in unerectile state (Figure 1(b)) [5].

**Figure 2:**
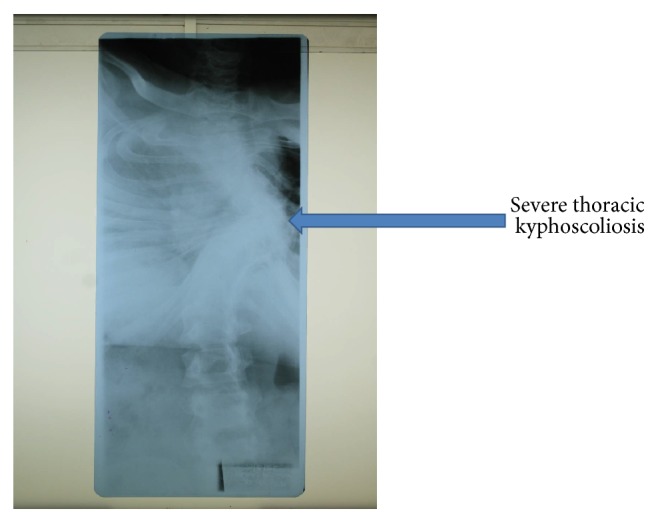
Chest X-ray showed severe kyphoscoliosis in a 25-year-old male with Prader-Willi Syndrome.

Chest X-ray revealed severe deformed cervical and thoracic spine, reduced lung volume with normal vasculature, and normal heart size. It also showed rotated thorax with severe kyphoscoliosis (Figure 2). Echocardiography and electrocardiography were normal. Abdominal ultrasound revealed normal kidneys, urinary bladder, liver, spleen, and gall bladder, but testes could not be visualized. Baseline blood investigations including full blood picture, blood sugar, electrolytes, thyroid hormone, lipids, renal, and liver function tests were all within normal range.

The clinical diagnosis was PWS. It was concluded that his respiratory failure was due to obesity and kyphoscoliosis. He was managed with reduced caloric intake, salbutamol, and prednisolone in combination with oxygen therapy. He kept on improving until the fifth week when he was nursed off oxygen. The patient was successfully discharged home with salbutamol, low carbohydrate, and vitamin enriched diet in week seven with a weight of 50 kg (BMI 28.7 kg/m^2^). The family members were instructed to monitor his diet and encourage physical exercise to at least maintain his discharge weight. When seen again after 3 months, his weight was 46 kg (BMI 26.4 kg/m^2^). Thereafter he was lost to follow-up.

Venous blood was sampled and sent to the Genome Diagnostic Laboratory, Department of Clinical Genetics, Maastricht University Medical Centre, Maastricht, Netherlands, for confirmation of the clinical diagnosis. The methylation specific MLPA analysis (MS-MLPA kit ME028-B2, MRC, Holland) showed the absence of the paternal allele due to a classical type 1 deletion of the Prader-Willi associated region 15q11–15q13, thereby confirming the clinical diagnosis of PWS.

## 3. Discussion

Clinical assessment strongly suggested a diagnosis of PWS. DNA methylation analysis confirmed this. Respiratory complications are frequently seen in PWS and are the most important cause of morbidity and mortality [1]. Respiratory failure due to kyphoscoliosis and aggravated by obesity might ensue [6, 7].

Obesity in PWS is a consequence of hyperphagia which is due to a dysfunctional hypothalamic-pituitary axis, leading to voracious eating [2]. Sleep disorders, like excessive sleeping and obstructive sleep apnoea (OSA), are another type of problem seen in PWS [6]. PWS patients have poor muscle coordination and poor gag reflex. This is also accompanied with production of very thick saliva. These characteristics together with obesity and kyphoscoliosis play a key role in respiratory failure. Management of PWS requires a holistic approach and includes medical, surgical, educational, and behavioural expertise, provided in a multidisciplinary team [6]. Medical management of patients with PWS includes control of blood sugar as well as cholesterol with hypoglycaemic and lipid lowering agents when necessary. Growth hormone treatment plays a significant role in the management when started during childhood. It also involves control of caloric intake to less than 1000 kcal/day and an increase of physical activities in order to reduce obesity and improve quality of life [6]. Psychosocial support for the family and patients is essential to motivate them to sustain these lifestyle changes. Controlled food intake, physical therapy, and regulated exercise can be potentially lifesaving [1, 6]. In our case we managed to achieve a significant reduction of his caloric intake and thereby his weight which helped to reduce his DIB. His DIB prohibited even light exercises during admission. The kyphoscoliosis seemed almost too advanced to be corrected surgically, apart from the fact that his pulmonary condition did not allow for such an intervention. Family members were educated to support him in dieting in order to avoid rebound of weight gain and recurrence of related respiratory problems. We also encouraged physical exercise but on follow-up after 3 months it appeared that only very light exercises were possible. Nonetheless, his weight went further down.

Surgical intervention is not of much use in correcting obstructive sleep apnoea (OSA). Patients underwent adenoidectomy, adenotonsillectomy, and even uvulopalatopharyngoplasty to correct OSA but improvement appeared marginal [7]. Minigastric bypass in the treatment of morbid obesity proved to bring significant weight reduction [6]; however laparoscopic sleeve gastrectomy is the better option nowadays. Careful judgment of type and amount of anaesthesia to be used during surgical management of PWS cases, particularly those with an already compromised respiratory system, is needed [7].

PWS patients are better off in special schools or schools with special needs and remedial teachers because of their cognitive impairment and behavioural problems [3]. It is well known that skin picking, temper tantrums, obsessive-compulsive features, and stealing are more prevalent among PWS children compared to other patients with intellectual disability [1]. Our patient dropped from school at the age of seven years because of on and off fighting with other children. Educational, psychosocial, and community workers need to be involved to work in collaboration with the medical team and family members. Before discharging our patient we had a group discussion between family members and the medical team, stressing necessary lifestyle changes and medical follow-up.

Though many cases of PWS have been reported globally, the few reported from Sub-Saharan countries were mostly from South Africa, but also one from Nigeria [8]. As far as we know, this is the first molecularly confirmed case to be reported from Sub-Saharan Africa outside South Africa. We stress the importance of suspecting PWS when there is a history of neonatal hypotonia, poor sucking, feeding problems during the first months of life and infancy, and later on features like short stature, hypogenitalism, hyperphagia, obesity, small hands and feet, kyphoscoliosis, respiratory problems, intellectual disability and behavioural problems developing. Molecular diagnosis of genetic diseases is in large parts of Africa not yet established. However, it is important to get confirmation of tentative clinical diagnoses through international collaboration, thereby allowing for patients and their families to have proper management, including genetic counselling.

In conclusion, we presented a case of respiratory failure as a complication of extreme obesity and kyphoscoliosis in a patient with molecularly confirmed PWS from a referral hospital in Northern Tanzania.
